# Longitudinal Changes in Depression, Anxiety and Stress Symptoms Among Hemodialysis Patients

**DOI:** 10.3390/clinpract16020037

**Published:** 2026-02-08

**Authors:** Adriana-Luciana Luca, Felicia Militaru, Mădălina Iuliana Mușat, Ion Udriștoiu, Eugen Moța

**Affiliations:** 1 U.M.F. Doctoral School Craiova, University of Medicine and Pharmacy of Craiova, 200349 Craiova, Romania; eugenmota@yahoo.com; 2 Department of Psychiatry, University of Medicine and Pharmacy of Craiova, 200349 Craiova, Romania; felicia.militaru@umfcv.ro (F.M.); ion.udristoiu@umfcv.ro (I.U.); 3 Experimental Research Centre for Normal and Pathological Aging, University of Medicine and Pharmacy of Craiova, 200349 Craiova, Romania; madalina.musat@umfcv.ro; 4 Department of Scientific Research Methodology, University of Medicine and Pharmacy of Craiova, 200349 Craiova, Romania

**Keywords:** chronic kidney disease, hemodialysis, comorbidity, depression, anxiety, stress

## Abstract

**Background/Objectives**: Chronic kidney disease (CKD) progresses with the gradual and irreversible loss of renal function. In Romania, given the increasing number of patients undergoing hemodialysis (HD), the prevalence of psychiatric symptoms and disorders in this population has become particularly significant. Although important advances have been made in the management of psychiatric conditions in HD patients, their mental health remains relatively poor. The aim of this study was to observe the severity temporal trends of depression, anxiety and stress symptoms and correlations among HD patients. **Methods**: A total of 173 patients, underwent a detailed anamnesis, with emphasis dialysis duration, comorbidities and a complex psychiatric evaluation, followed by the application of the Socio-economic Scale (SES-3); Mini Mental State Examination (MMSE); and the Depression, Anxiety and Stress Scale 21R (DASS-21R). The dialysis performance (spKt/V) and Charlson Comorbidity (CCI) indices were provided by DIAVERUM Nephrology and Dialysis Center in Craiova. **Results**: The severity of depression and anxiety symptoms significantly increased over six months, 0.248 ± 1.432 vs. 0.453 ± 1.488 (*p* < 0.0001; r_rb_ = 0.296) for depression, and −0.090 ± 1.004 vs. 0.089 ± 1.047 (*p* < 0.0001; r_rb_ = 0.252) for anxiety; while stress-like symptoms remained stable 0.080 ± 1.318 vs. 0.164 ± 1.357 (*p* = 0.0661; r_rb_ = 0.123), despite improvements in dialysis adequacy (spKt/V). Depression scores were moderately correlated with anxiety and weakly correlated with stress and spKt/V. Anxiety results were moderately correlated with stress, while both anxiety and stress showed negligible correlations with spKt/V. Clinical variables assessed showed moderate predictive value for psychological outcomes in this cohort. **Conclusions**: Our study confirms the temporal trend of severity of mental symptoms and their persistence among HD patients, highlighting the urge to integrate mental health screening and intervention programs and a multidisciplinary team adapted for each case.

## 1. Introduction

Chronic kidney disease (CKD) progresses towards the gradual and irreversible loss of renal functions, ultimately requiring renal replacement therapy [1].

During its progressive course, CKD is often associated with various somatic complications, such as arterial hypertension, left ventricular hypertrophy and uremic pericarditis; metabolic and nutritional changes; skeletal alterations; hematological complications including anemia; chronic inflammation; uremic encephalopathy; and, importantly, various degrees of psychiatric impairment [2,3,4].

In the final stage of CKD, dialysis becomes essential for patient survival. However, it imposes numerous restrictions: a strict dialysis schedule and hygiene–dietary limitations, triggering global changes in the patient, affecting physical, social and professional domains, with significant repercussions on mental health [5,6,7,8].

Regarding prevalence of mental health perturbances in hemodialysis (HD) patients, the most common alterations are depression and anxiety, reduced affective display, fatigue, feelings of incurability, feelings of uselessness, and anhedonia [9,10]. These conditions lead to decreased global functioning and an increased risk of suicide [11].

The recent literature highlights this burden in cross-sectional studies. For example, a 2021 study conducted on 524 HD patients from Sharkia Governorate, Egypt, using the Hospital Anxiety and Depression Scale (HADS), reported depression in 31.9% and anxiety in 33.7% of participants [12]. In 2020, another cross-sectional study from Egypt involving 117 HD patients, assessing Structured Clinical Interview for DSM Disorders (SCIDI) found an extremely high prevalence of psychiatric disorders (75.21%), with depression reported in 56.4% and anxiety in 51.3% [13].

Long-term studies indicate that these symptoms are not only highly prevalent but also relatively stable over time. In a 12-month prospective study including both recent and ongoing HD patients, Ng et al. identified individuals with persistently elevated levels of depression and anxiety, alongside subgroups with new-onset or remitting symptoms, pointing to heterogeneous emotional trajectories [14]. Likewise, a 16-month follow-up by Cukor et al. showed that diagnoses of depression and anxiety tend to persist and are associated with declines in health status [15].

Several other longitudinal cohorts have demonstrated an association between depressive symptoms and clinical outcomes. Both repeated assessments of depression in HD patients and baseline scores on psychometric instruments have been linked to increased mortality, with effect sizes comparable to those of established medical risk factors [16,17,18].

Although significant progress has been made through rapid psychotherapeutic interventions, counseling and psychiatric treatment, much more remains to be achieved [19,20,21]. Psychosocial interventions may partially modify the trajectories of anxiety and depression over a 12-month period; however, a subset of patients continues to follow “high-stable” symptom courses [22].

Cross-sectional evidence from Eastern Europe consistently shows a high prevalence of depression and anxiety among HD patients, with findings from Poland, Croatia, and Romania indicating substantial affective burden influenced by demographic and treatment-related factors [23,24,25,26]. Additional studies emphasize associations between stress and depressive symptoms and highlight the risk of underdiagnosing depression when relying solely on screening instruments [24,25].

To the best of our knowledge, there are no longitudinal studies from Eastern Europe that have repeatedly assessed mental health symptoms in HD patients. Additionally, psychological stress is seldom assessed as a distinct construct in longitudinal studies, and instruments that simultaneously quantify depression, anxiety, and stress have been used almost exclusively in cross-sectional or short-term interventional research among dialysis patients [27]. These gaps limit understanding of short-term symptom dynamics, supporting the relevance of a six-month observational window. A six-month interval is clinically meaningful in routine dialysis care, as it captures symptom evolution beyond transient fluctuations while remaining feasible for repeated assessment in medically burdened patients.

While longitudinal assessments of affective symptoms in HD populations have been reported previously, evidence remains limited for short-term trajectories and for underrepresented Eastern European cohorts. The present study builds incrementally on existing designs by applying a culturally adapted instrument and by examining depression, anxiety, and stress as distinct symptom dimensions over a six-month period. To address these gaps, this prospective observational longitudinal cohort study aimed as primary objective to assess the trajectories of depression, anxiety and stress symptoms among HD patients at the DIAVERUM Nephrology and Dialysis Center in Craiova, Romania using Depression, Anxiety, Stress Scale 21R (DASS-21R) adapted and standardized for the Romanian population, to find correlations between severity of mental health symptoms and clinical and paraclinical aspects—Charlson Comorbidity Index (CCI) and dialysis performance index (spKt/V), and to recognize the urgent need for rapid intervention and multidisciplinary collaboration. The novelty of the present study lies in its regional and methodological specificity. First, it focuses on a Romanian cohort of HD patients, an underrepresented population in Eastern European longitudinal mental health research. Second, it applies the Romanian-adapted and standardized version of the DASS-21R in a longitudinal HD context, extending its use beyond cross-sectional designs. Third, it examines changes in depression, anxiety, and stress symptom severity over a six-month interval, a timeframe that has been less frequently explored in previous longitudinal studies of dialysis populations. Together, these aspects may contribute to a more context-sensitive understanding of emotional symptom trajectories in HD patients and may highlight the potential relevance of timely psychological assessment and referral pathways in HD care.

## 2. Materials and Methods

### 2.1. Study Design and Subjects

This prospective, longitudinal, observational, single-center cohort study was conducted at the DIAVERUM Nephrology and Dialysis Center in Craiova over a two-year period, from December 2021 to November 2023. It included patients with end-stage CKD who had been undergoing HD for at least one year, three times per week. We considered a one-year period to be sufficient for patients to adapt to the HD regimen.

The initial study cohort consisted of 220 participants. We excluded 7 patients who presented MMSE scores ≤ 20 who had severe cognitive impairment; 6 patients with depressive disorder under psychiatric treatment, 1 patient with schizophrenia in his medical records; 1 patient with moderate to severe intellectual disability and 1 patient with congenital deafness who had difficulty understanding and responding to the questionnaires; 17 patients with major inconsistent responses between the two applications of the instruments whose data were considered unreliable; 9 patients who decided to voluntarily discontinue participation because of the fatigue; and 5 patients who died during the study period.

**Figure 1:**
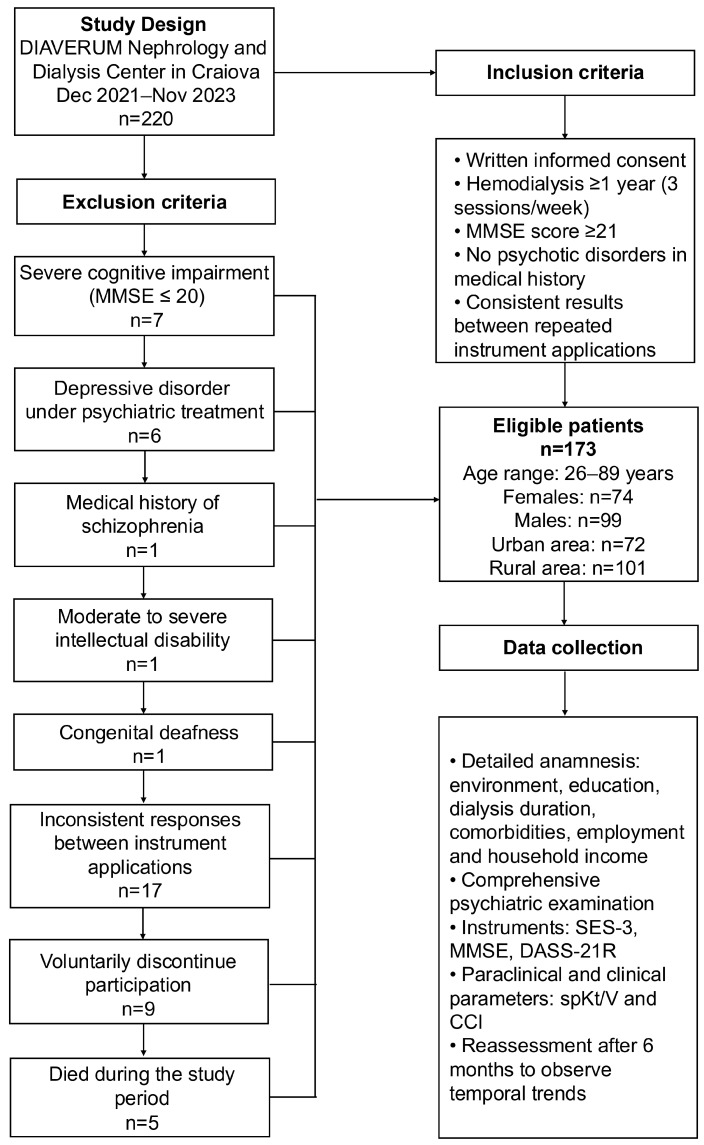
Study design and data collection process. Flowchart illustrating study period, inclusion and exclusion criteria, sample characteristics and data collection methods applied to 173 HD patients (74 females, 99 males; age range: 26–89 years) at the DIAVERUM Nephrology and Dialysis Center in Craiova (December 2021–November 2023) (created using Adobe InDesign 21.0.1).

The final study cohort consisted of 173 patients—74 women and 99 men—with ages ranging from 26 to 89 years. Of these, 72 were from urban areas and 101 from rural areas (Figure 1).

### 2.2. Inclusion Criteria

Inclusion criteria included patients with end-stage CKD who were able to understand and respond to the study questionnaires in Romanian, could provide written informed consent and had HD for at least one year with a frequency of three times per week.

### 2.3. Exclusion Criteria

Exclusion criteria included patient choice of withdrawal from the study, an MMSE score ≤ 20, presence of medical comorbidities or psychiatric disorders in medical history or recent diagnosis at the time of the research that would interfere with the ability to understand and respond to the research instruments, undertaking psychotropic medication and inconsistent results between the two instrument applications.

### 2.4. Data Collection Process

After obtaining written informed consent from each patient, data collection began, including the following:

- A detailed anamnesis emphasizing environment of origin, educational level, dialysis duration, associated comorbidities, occupation and monthly household income.
- A comprehensive psychiatric examination assessing the entire mental status and recording the full range of symptoms.
- Administration of the Socio-economic Scale (SES-3); Mini Mental State Examination (MMSE); and the DASS-21R adapted and standardized for the Romanian population.
- The DIAVERUM Nephrology and Dialysis Center in Craiova provided access to relevant statistical data for this study, such as spKt/V and CCI.

These instruments (except SES-3), and clinical parameters were applied and noted twice: at baseline (T1) and after six months (T2) to minimize response variability and to observe changes in mental health status (Figure 1). This was an observational study and no study-mandated psychological intervention was delivered between T1 and T2; participants continued their usual HD care.

To ensure the validity and reliability of the self-reported data, we implemented a response-quality screening procedure. Inconsistent responses were defined as patterns indicating low engagement with the questionnaire rather than true symptom reporting. Specifically, excluded participants showed response profiles suggestive of superficial or non-attentive completion of the DASS-21R (*n* = 17). Participants were excluded if they showed:

(1) logically contradictory answers across semantically similar items (e.g., endorsing both extreme absence and extreme presence of the same symptom construct), and/or
(2) large, random-like fluctuations across items within the same subscale that were incompatible with a coherent symptom profile
(3) substantial item non-response, defined as refusal or failure to answer multiple items, resulting in incomplete response profiles.

This approach is consistent with established methodological guidelines for questionnaire-based research and aims to minimize measurement error [28,29,30,31].

### 2.5. Variables and Measures

Cognitive deficits were assessed using the MMSE, first introduced by Dr. Marshall Folstein in 1975 [32]. The MMSE evaluates temporal–spatial orientation, attention, immediate and short-term memory, ability to perform concrete–abstract executive tasks, motor skills and language. It consists of 11 items, with total score ranging from 0 to 30: 0 indicates severe cognitive decline requiring institutionalization, while 30 indicates normal cognitive functioning [33].

Negative emotional symptoms of depression, anxiety and stress and their severity were assessed using DASS-21R, a self-report psychometric screening instrument developed in 1995 by Syd Lovibond and Peter Lovibond as a shortened version of DASS-42, which was adapted and standardized for the population in Romania under the coordination of Adela Perțe and Monica Barbu [34]. It includes 3 subscales with 7 items each, rated from a 0 to 3 Likert scale: 0—did not apply to me at all, 1—applied to me to some degree, or some of the time, 2—applied to me to a considerable degree, or a good part of the time, 3—applied to me very much, or most of the time.

The depression scale assessed (1) dysphoria, (2) hopelessness, (3) anhedonia, (4) apathy–abulia, (5) inactivity, (6) life devaluation and (7) self-esteem. The anxiety scale assessed (1) autonomic arousal, (2) musculoskeletal effects, (3) situational anxiety and (4) subjective anxiety experience. The stress scale, sensitive to chronic nonspecific arousal, assessed (1) psychological tension, (2) irritability and (3) hyperactivity.

For each subscale, a raw score of up to 21 was obtained. The raw score for each participant was then converted into a sex- and age (10-year intervals)-adjusted z-score according to the DASS-21R manual. Based on the resulting *z*-score, severity levels of symptoms were defined as follows: normal ≤ 0.5; mild 0.5 < z < 1.0; moderate 1.0 ≤ z < 2.0; severe 2.0 ≤ z < 3.0; extremely severe ≥ 3.0 [35].

We standardized all DASS-21R raw scores to z-scores to ensure comparability between time points. Raw scores often differ in variance across assessments; therefore, z-scores transformation provides a reduced distribution bias, and allows more valid detection of changes in symptom severity.

Socio-economic status was assessed at baseline using the SES-3 composite index (education, occupation, and household income), with total scores ranging from 0 to 6 and categorized as low (0–2), medium (3–4), or high (5–6) [36,37,38].

The effectiveness of a HD session is given by spKt/V, an essential parameter in CKD treatment [39]. The spKt/V values were provided by the DIAVERUM Nephrology and Dialysis Center in Craiova. The target values were as follows: <1.2 = inadequate (increased risk of complications and mortality if dialysis is not adjusted); 1.2–1.4 = adequate; >1.4 = optimal target (no additional proven clinical benefit beyond 1.6–1.8; higher values increase risks of hypotension, high catabolism and unnecessary resource use).

Long-term mortality risk was estimated by the DIAVERUM Nephrology and Dialysis Center in Craiova using the CCI, developed by Dr. Mary Charlson and colleagues in 1987 [40,41]. The CCI evaluates 19 categories of comorbidities, assigning scores based on severity. The sum represents the patient’s mortality risk [42]: 0 = no comorbidities; 1–2 = low comorbidity; 3–4 = moderate comorbidity; ≥5 = high comorbidity, increased mortality risk.

### 2.6. Data Analysis

Statistical data analysis was performed using IBM SPSS Statistics version 26 software (IBM Corp., Armonk, NY, USA), GraphPad 10.3.1 (GraphPad Software, Inc., San Diego, CA, USA), and Microsoft Office 365 (Microsoft Corp., Redmond, WA, USA). The figures were created using Adobe InDesign 21.0.1 (Adobe, San Jose, CA, USA). Demographic characteristics (gender, age categories, environment of residence, level of education, and household income thresholds) were summarized using absolute frequencies and percentages (*n*/%). Socio-economic status components, including educational attainment, occupational category, and household income were similarly described, using SES-3 composite method. Data normality was assessed using D’Agostino & Pearson test and Shapiro–Wilk test. As the data did not follow a normal distribution, T1–T2 differences were analyzed using the nonparametric Wilcoxon signed-rank test for paired observations. Effect size was calculated using the rank-biserial correlation (r_rb_) specific to the Wilcoxon signed-rank test [43]. Correlations between the analyzed parameters were examined using Spearman’s rank correlation coefficient, with significance assessed using two-tailed *p*-values. Multivariate logistic regression analyses were performed to identify independent predictors of depression, anxiety, and stress. The predictor variables included age (years), sex, CCI), and years on dialysis. Model performance was evaluated using receiver operating characteristic (ROC) curve analysis, with the area under the curve (AUC) used as an indicator of discriminatory ability. The threshold used to define the presence of depression, anxiety, or stress symptoms was a DASS-21R z-score of 0.5. Scores ≤ 0.5 were coded as 0 (absence of symptoms, normal range), while scores > 0.5 (mild, moderate, severe, or extremely severe) were coded as 1 (presence of symptoms). Odds ratios (ORs) with corresponding 95% confidence intervals (CI) were calculated for each predictor. Statistical significance was set at α = 0.05. Data are presented as percent or mean ± standard deviation (SD), and statistical significance is indicated as follows: * *p* < 0.05, ** *p* < 0.01, *** *p* < 0.001, **** *p* < 0.0001.

### 2.7. Research Ethics

This study was conducted in accordance with the Declaration of Helsinki and approved by the Ethics Committee of the University of Medicine and Pharmacy of Craiova, no. 177/29.10.2021, with all patients agreeing to participate. At the end of the study, participants who screened with severe symptom levels on any DASS-21R subscale (z ≥ 2.0; severe including extremely severe) were informed and offered referral for clinical evaluation through standard institutional care pathways, consistent with evidence-based guidance for depression, anxiety, and stress management [44,45,46].

## 3. Results

Among the 173 patients included in this study, 42.8% were women (*n* = 74) and 57.2% men (*n* = 99). We conducted a power analysis using the mean and standard deviation of the variables and evaluated statistical power for a target value of 0.8, which is a commonly accepted standard in research, with a two-tailed alpha level of 0.05. The results indicated that the minimum required sample size for detecting differences in depression scores was 44 patients, while for anxiety, stress, and spKt/V, the required sample size was 53 patients. In our study, the number of participants exceeded these minimum requirements, ensuring adequate statistical power to detect meaningful differences between groups.

By age distribution, 1.2% patients were ≤29 years old, 4% were between 30 and 39, 15% between 40 and 49, 25.4% between 50 and 59, 28.9% between 60 and 69, 20.8% between 70 and 79, and 4.6% ≥80 (Table 1), with a mean age of 61.19 ± 11.54 years for female, and 60.03 ± 13.29 years for male.

### 3.1. SES-3 Descriptive Results

Overall, most participants had high school/post-secondary/vocational education (51.4%, *n* = 89), while 31.2% (*n* = 54) had primary/middle school education and 17.3% (*n* = 30) had university/postgraduate studies. Occupationally, 46.2% (*n* = 80) were unskilled/manual/agricultural/unemployed, 35.8% (*n* = 62) were administrative/services/sales/technicians, and 17.9% (*n* = 31) were professionals/entrepreneurs/managers. Household income was above the national median threshold in 69.9% (*n* = 121) of participants, while 30.1% (*n* = 52) were in the 50–100% median threshold category. Using the SES-3 composite, medium socio-economic status predominated (49.7%, *n* = 86), followed by low (31.8%, *n* = 55) and high (18.5%, *n* = 32); the distribution by sex is shown in Table 2. Low SES was more frequent among females (44.6%) than males (22.2%), whereas high SES was more frequent among males (22.2%) than females (13.5%).

### 3.2. None of the Participants Showed Quantifiable Cognitive Impairment

The MMSE score was ≥21 in all patients, and variations between the two assessments (27.20 ± 2.35 and 27.64 ± 2.40) were minimal, within the inclusion criteria.

### 3.3. Depression and Anxiety Severity Scores, Together with Dialysis Adequacy, Increased from T1 to T2, Whereas Stress Severity Levels Remained Stable Across Time Points

**Figure 2:**
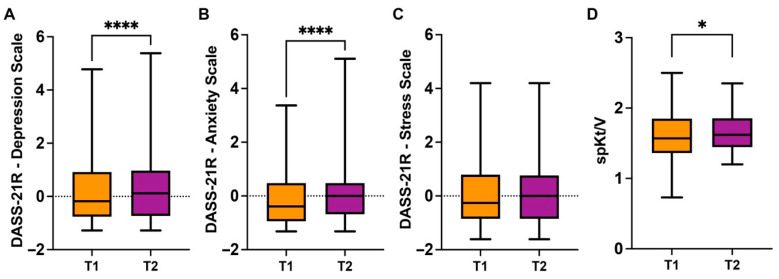
Changes in psychological and clinical measures between Time 1 (T1) and Time 2 (T2). (**A**) DASS-21R Depression severity scores at T1 and T2. (**B**) DASS-21R Anxiety severity scores at T1 and T2. (**C**) DASS-21R Stress severity scores at T1 and T2. (**D**) spKt/V values at T1 and T2. Significance levels are marked as follows: * *p* < 0.05, **** *p* < 0.0001.

Analysis using the Wilcoxon signed-rank test showed elevated DASS-21R Depression severity scores at T2 (0.453 ± 1.488) compared to T1 (0.248 ± 1.432) (*p* < 0.0001; r_rb_ = 0.296) (Figure 2A).

To improve clinical interpretability, we summarized the distribution of DASS-21R severity categories at both assessments. At T1 vs. T2, depression was classified as absent in 69.9% vs. 69.9%, mild in 6.4% vs. 5.8%, moderate in 10.4% vs. 11.0%, and severe (including extremely severe) in 13.3% vs. 13.3% of participants. Anxiety was absent in 78.6% vs. 79.2%, mild in 8.1% vs. 7.5%, moderate in 8.1% vs. 8.1%, and severe (including extremely severe) in 5.2% vs. 5.2%. Stress was absent in 69.9% vs. 70.5%, mild in 8.7% vs. 8.1%, moderate in 11.0% vs. 11.0%, and severe (including extremely severe) in 10.4% vs. 10.4%. Category shifts over six months were rate: for depression, 1 patient (0.6%) worsened and 1 (0.6%) improved, while 171 (98.8%) remained in the same severity range; for anxiety and stress, no patient worsened and 1 (0.6%) improved for each scale, with 172 (99.4%) remaining stable.

As part of the study’s ethical safeguards, 22.5% participants (*n* = 39) met the severe threshold (z ≥ 2.0 on at least one DASS-21R subscale) at both assessments and were offered at the end of the study referral for clinical evaluation through standard care pathways.

A similar pattern was observed for the DASS-21R Anxiety scale (Figure 2B), where T2 severity values (0.089 ± 1.047) were also increased compared to T1 (−0.090 ± 1.004) (*p* < 0.0001; r_rb_ = 0.252). In contrast, no difference was found between T1 (0.080 ± 1.318) and T2 (0.164 ± 1.357) on the DASS-21R Stress scale (Figure 2C; *p* = 0.0661; r_rb_ = 0.123).

For the clinical parameter spKt/V (Figure 2D), T2 values (1.645 ± 0.271) were higher than T1 values (1.595 ± 0.342) (*p* = 0.0103; r_rb_ = 0.193).

### 3.4. Correlational Analysis of Psychological Outcomes and Dialysis Adequacy

**Figure 3:**
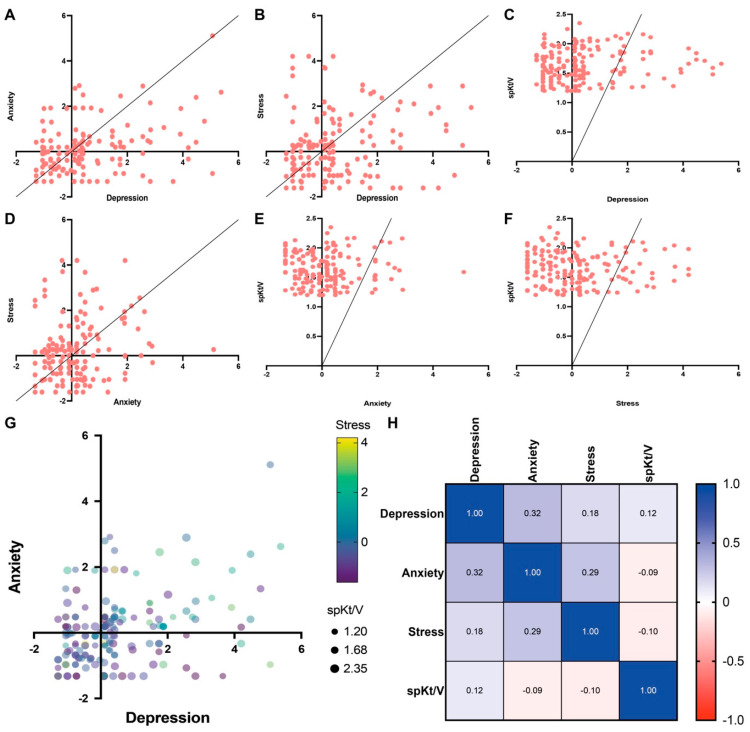
Correlations among psychological measures (Depression, Anxiety, Stress) and dialysis adequacy (spKt/V). (**A**) Scatterplot showing the relationship between Depression and Anxiety severity scores. (**B**) Scatterplot showing the relationship between Depression and Stress severity scores. (**C**) Scatterplot showing the relationship between Depression and spKt/V. (**D**) Scatterplot showing the relationship between Anxiety and Stress severity scores. (**E**) Scatterplot showing the relationship between Anxiety and spKt/V. (**F**) Scatterplot showing the relationship between Stress and spKt/V. (**G**) Combined scatterplot illustrating the association between Depression and Anxiety, with point color indicating Stress levels and point size representing spKt/V. (**H**) Heatmap displaying Spearman correlation coefficients (Spearman r) among Depression, Anxiety, Stress, and spKt/V.

Depression was moderately correlated with anxiety (r = 0.32) (Figure 3A) and weakly correlated with stress (r = 0.18) (Figure 3B) and spKt/V (r = 0.12) (Figure 3C). Anxiety was moderately correlated with stress (r = 0.29) (Figure 3D), while both anxiety and stress showed negligible correlations with spKt/V (r = −0.09 and r = −0.10, respectively) (Figure 3E,F).

A combined visualization (Figure 3G) further highlights the clustering of depression and anxiety severity values, while also showing variability in stress levels (color-coded) and dialysis adequacy (point size). The correlation matrix (Figure 3H) confirmed these patterns (Table 3).

Table 4 summarizes the relationships between psychological variables and clinical parameters. Depression, anxiety, and stress severity levels showed no meaningful associations with comorbidity burden or dialysis duration, while spKt/V showed a small negative association with CCI.

### 3.5. Clinical Variables Show Moderate Predictive Value for Psychological Outcomes in This Cohort

**Figure 4:**
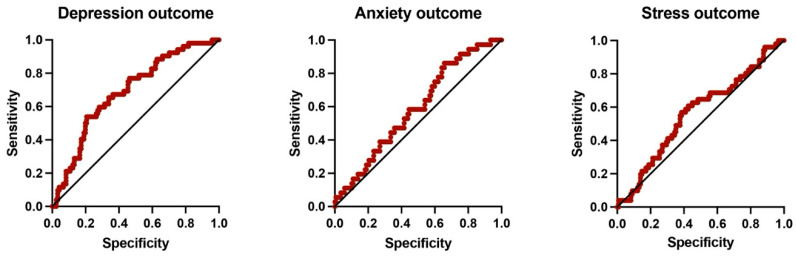
Receiver operating characteristic (ROC) curves illustrating the discriminatory performance of the multivariate logistic regression models for depression, anxiety, and stress. Each model included age, sex, CCI, and years on dialysis as predictors. Each panel shows a ROC curve (red line) and the reference line (black diagonal).

Multivariate logistic regression analyses were conducted to identify independent predictors of depression, anxiety, and stress. The predictors included age, sex, CCI, and years on dialysis (Figure 4).

Across all three outcomes, the models demonstrated limited explanatory power, and wide confidence intervals across predictors. Together, these findings suggest that age, sex, comorbidity index, and dialysis vintage have moderate predictive power of the included clinical variables for psychologic outcomes in this cohort (Table 5).

## 4. Discussion

The results of the present study provide new evidence regarding temporal changes in severity of depression, anxiety, and stress symptoms among Romanian HD patients, which can be observed using the standardized DASS-21R self-report questionnaire, adapted for the country’s population.

The use of the DASS-21R psychometric research instrument allows for the simultaneous assessment of three major mental health dimensions—depression, anxiety and stress. Additionally, being an easy-to-administer, reliable and valid instrument, it proved suitable for this category of patients, whose fatigue and the significant time required for dialysis treatment may limit their availability for complex psychological assessments.

Compared with other frequently used instruments, such as the State Trait Anxiety Inventory (STAI), Hospital Anxiety and Depression Scale (HADS), or Beck Depression Inventory (BDI), DASS-21R has the advantage of integrating stress as a distinct construct, which is particularly relevant in the context of adaptation to prolonged treatment for chronic illness [47].

In addition, a number of longitudinal studies in chronic disease populations have used the DASS-21 to monitor changes in emotional symptoms over time, supporting its utility in capturing symptom trajectories rather than diagnosing mental disorders [48,49,50,51,52]. This aligns with our study’s objective of observing fluctuations in depression, anxiety, and stress-related symptoms rather than establishing psychiatric diagnoses.

However, these results warrant cautious interpretation, as the study did not include a structured clinical interview to formally establish psychiatric diagnoses.

Our findings indicate the severity of depression and anxiety symptoms increased over six months, from 0.248 ± 1.432 in T1 to 0.453 ± 1.488 in T2 (*p* < 0.0001; r_rb_ = 0.296) for depression, and from −0.090 ± 1.004 in T1 to 0.089 ± 1.047 in T2 (*p* < 0.0001; r_rb_ = 0.252) for anxiety; whereas stress-like symptoms remained stable 0.080 ± 1.318 in T1 and 0.164 ± 1.357 in T2 (*p* = 0.0661; r_rb_ = 0.123), despite improvements in dialysis adequacy (spKt/V), with T2 values (1.645 ± 0.271) higher than T1 values (1.595 ± 0.342) (*p* = 0.0103; r_rb_ = 0.193), suggesting that stress might be perceived as chronical prolonged symptom that is independent from depressive or anxiety symptoms, or the studied clinical and paraclinical parameters.

Clinically, the worsening of depression and anxiety over a relatively short timeframe may be driven by cumulative treatment fatigue, persistent symptom burden (such as pruritus, insomnia, pain), but also by psychosocial factors that have been shown to influence emotional trajectories in longitudinal studies of HD patients [53,54,55,56]. Previous cohort studies have identified low perceived social support, maladaptive coping strategies, and illness-related uncertainty as key determinants of “high-stable” or worsening symptom trajectories over time, whereas stronger social support and adaptive coping are associated with more favorable emotional courses [57,58,59,60,61,62]. Although these psychosocial variables were not directly measured in the present study, their established role provides a relevant interpretative framework for the observed symptom progression.

Beyond psychosocial mechanisms, longitudinal evidence from other cohorts suggests that systemic inflammation may contribute to depressive symptom trajectories in HD patients. Elevated inflammatory markers, particularly C-reactive protein and interleukin-6, have been associated with persistent or worsening depression over time, independent of dialysis adequacy and comorbidity burden [16,53,63,64,65]. These findings support a biopsychosocial framework in which inflammatory processes may sustain emotional distress in a subset of patients. As inflammatory biomarkers were not assessed in the present study, this biological pathway could not be examined directly.

These observations support previous longitudinal findings showing that affective symptoms in HD patients tend to remain persistent or worsen over time. For instance, Ng et al. reported increases in depression and anxiety of approximately 0.40–0.60 SD over 12 months [14], while the changes in our sample correspond to ~0.20 SD for depression and ~0.18 SD for anxiety, indicating a milder, yet directionally similar pattern. Likewise, Cukor et al. found that over 70% of HD patients exhibited persistent or worsening affective symptoms during a 16-month period [15]. The incremental worsening of depression and anxiety symptoms in our cohort fits within a worsening-stable trajectory, consistent with international findings.

Moreover, repeatedly elevated depressive symptoms have been strongly associated with increased mortality in HD outpatients [16,17,18], highlighting the clinical relevance of our results.

Although statistically significant, the effect sizes in our study were small (depression r_rb_ = 0.296, anxiety r_rb_ = 0.252, stress r_rb_ = 0.123). These values fall within the expected range of naturalistic symptom fluctuations documented in dialysis populations, which typically show small-to-moderate effect sizes over time [14,15].

The categorical analysis of DASS-21R severity provides important clinical context for the statistically significant mean changes observed over six months. Despite small increases in mean depression and anxiety scores, the vast majority of patients remained within the same severity category across assessments, with over 98% showing no categorical change. This finding suggests that, at the group level, symptom progression over a six-month interval is generally modest and characterized more by stability than by clinically meaningful deterioration.

At the same time, a substantial subgroup of patients consistently met criteria for severe symptoms on at least one emotional dimension, highlighting the presence of persistent high-burden cases that may not be fully captured by mean score changes alone. These results underscore the importance of complementing continuous symptom metrics with categorical severity distributions when interpreting longitudinal mental health data in HD populations.

In terms of values, our standardized DASS-21R z-scores are comparable to those reported in European studies using HADS or BDI, where most patients fall within the normal–mild symptom range but with wide interindividual variability [23,24,25,66].

The stability of stress symptoms despite increases in depression and anxiety levels mirrors the partial independence of DASS-21 subscales, as shown by Lovibond and Lovibond and later Romanian validation studies [34].

Depression, anxiety and stress severity symptoms in our cohort showed no associations with dialysis adequacy (*r* = 0.1157, *r* = −0.08782, *r* = −0.09958), comorbidity burden (*r* = 0.05526, *r* = 0.05052, *r* = −0.01321), or dialysis duration (*r* = −0.06729, *r* = −0.01855, *r* = −0.1495), similar to previous research demonstrating that emotional distress in HD patients is probably driven by psychosocial rather than biological factors [19,20,21]. Even though spKt/V improved, this did not translate into better mental health outcomes, confirming evidence that optimizing dialysis dose alone does not mitigate depression or anxiety symptoms [39]. The CCI summarizes the burden of 19 major comorbid conditions into a single weighted score reflecting overall mortality risk. In clinical terms, scores of 0 indicate absence of relevant comorbidity, values of 1–2 correspond to low comorbidity burden, 3–4 to moderate burden, and scores ≥ 5 denote high comorbidity associated with increased mortality risk.

Depression showed a moderate correlation with anxiety (r = 0.32) and a weak association with stress (r = 0.18), consistent with the typical internalizing symptom cluster commonly documented in dialysis populations [9,10,11].

The multivariate models showed only limited ability to discriminate between levels of severity symptoms of depression, anxiety, and stress, with AUC values around 0.6. This indicates that age, sex, comorbidity burden, and dialysis vintage have modest and inconsistent associations with mental health outcomes in this cohort, probably a consequence of single center analysis, underscoring the need for broader, patient-centered assessment approaches. This limited discriminative ability is consistent with the use of a low clinical threshold capturing mild symptoms, which increases sensitivity at the expense of specificity.

A biopsychosocial conceptual framework may therefore be more appropriate, in which clinical and treatment-related factors interact with symptom burden, psychosocial resources (e.g., perceived social support and coping strategies), behavioral factors such as sleep quality, and biological pathways (e.g., systemic inflammation) to shape longitudinal trajectories of depression, anxiety, and stress.

These findings emphasize the importance of incorporating psychosocial assessments—such as social support, coping strategies, and illness perceptions—into future models, as these variables have shown stronger predictive value for emotional distress in chronic illness populations [60,67,68].

While the present study provides insight into short-term symptom trajectories, its single-center design and reliance on self-report measures limit diagnostic precision and generalizability. Future multicenter studies incorporating structured clinical interviews and broader psychosocial assessments may help clarify which modifiable factors most strongly shape mental health outcomes and how targeted interventions influence symptom trajectories over longer follow-up periods.

## 5. Limitations

Our study is subject to several limitations, which merit discussion and should be considered. For example, this research presented several sources of potential bias.

The DASS-21R is not a diagnostic tool but a psychometric screening instrument; therefore, the results must be interpreted with caution and correlated with psychiatric evaluations, including comprehensive clinical assessments [27].

A second limitation reported in the literature concerns the self-report nature of the scale, which may induce subjective response and recall bias. To minimize measurement bias, instruments were administered twice by the same trained evaluator, excluding participants who exhibited substantial variability in their responses between administrations, as recommended by psychometric guidelines. Although applied to improve data validity, the removal of participants with low-engagement response patterns could have reduced the representativeness of the sample by excluding individuals who were less motivated, more fatigued, or cognitively burdened during questionnaire completion. Such characteristics may themselves be associated with higher psychological distress. Therefore, this procedure limits the generalizability of the findings to more engaged respondents.

Nevertheless, the possibility of residual information bias remains, given the reliance on medical records and self-reported socio-economic data.

Attrition bias may also be present, given that some participants discontinued involvement or died during the six-months interval, potentially leaving a healthier or more resilient cohort at T2.

Several other limitations should be acknowledged. This study was conducted in a single dialysis center in Romania, which may introduce selection bias and limit the generalizability of the findings to other regions or populations. Also, while two assessments were performed six months apart, we did not include a long-term follow-up to evaluate the persistence or progression of severity of psychological symptoms or non-linear trajectories reported in multi-year cohorts [14,15]. In this regard, the six-month timeframe, although practical for routine clinical monitoring [63,69], may not capture the complex, dynamic emotional trajectories described in studies with extended follow-up intervals. Longer observation periods would allow clearer differentiation between transient fluctuations and persistent affective patterns with stronger prognostic implications.

As for validity, assessing psychological symptoms in HD patients requires particular attention, as somatic features of CKD may mimic depressive and anxiety symptoms. Using DASS-21R, a tool validated for the Romanian population that prioritizes mental health indicators over somatic ones, limiting clinical overlap. Measurement validity was further supported by a comprehensive psychiatric assessment and exclusion of cognitive impairment through MMSE screening. This integrated approach aligns with international recommendations and supports the reliability of mental health severity estimates obtained in our cohort. However, the use of validated self-report questionnaires without a structured clinical assessment introduces a risk of misclassification bias, as symptom severity may be over- or underestimated compared with clinician-administered diagnostic tools.

Finally, several psychosocial determinants known to shape emotional outcomes in CKD—such as perceived social support, illness perceptions, pain severity, sleep quality, or overall quality of life were not assessed. These factors are well-established contributors to emotional distress in CKD and their omission likely reduced the explanatory power of the statistical.

The predictive performance of the regression models was low (AUC values ranging from 0.56 to 0.68), indicating only modest discriminatory ability. Therefore, the limited predictive performance of regression models in our study suggests that unmeasured psychosocial variables—such as coping strategies, family support, and perceived illness burden—may play a more prominent role than clinical indicators alone in shaping emotional symptom burden among HD patients. Their inclusion in future work may substantially enhance the explanatory value of predictive models and provide a more accurate representation of psychological risk profiles in HD patients.

Taken together, these aspects underscore the need for future studies with extended follow-up intervals to integrate a broader spectrum of psychosocial, behavioral, and clinical variables to more accurately characterize the determinants of mental health trajectories in HD populations.

Despite the potential limitations that may persist, the obtained results underscore the urgent need to include routine mental health assessments, using instruments such as DASS-21R, in current nephrological practice, and prompt intervention.

## 6. Conclusions

In this cohort of HD patients, depressive and anxiety symptom severity showed statistically significant but small mean increases over the 6-month follow-up, whereas stress levels remained largely unchanged, even as dialysis adequacy improved. While these trajectories were modest in magnitude at the individual level, they may suggest a persistent psychological vulnerability in patients with CKD and indicate that symptom burden can evolve over time alongside routine clinical care.

In this context, routine mental health monitoring may be considered throughout dialysis care, using validated instruments. Implementing brief, structured screening tools—such as the DASS-21R at predetermined intervals—may facilitate the early recognition of emerging symptoms and help identify patients who may benefit from timely support or further assessment, particularly when clinically relevant distress is detected.

In addition, feasible supportive components delivered within dialysis settings could be explored, such as concise psychoeducational input (e.g., brief explanations of the interplay between chronic illness, stress and emotional symptoms, pain management, and treatment adherence; succinct educational materials; and early indicators for seeking professional support), resilience-oriented approaches (e.g., focused cognitive reframing, adaptive coping skills training, or brief mindfulness practices), and clearly defined referral pathways. Such elements may help address modifiable psychosocial contributors to emotional distress and support patients’ adaptive capacity, while acknowledging that the present data do not justify broad clinical recommendations.

Early identification of emotional distress, timely referral when indicated, and coordinated multidisciplinary input may be considered components of comprehensive HD management. Nevertheless, the current observational findings do not support strong claims regarding the independence of stress from other symptom dimensions. Future multicenter studies with larger and more diverse samples, ideally incorporating structured clinical interviews and longer follow-up, are warranted to better delineate determinants of mental health trajectories in this population and to evaluate targeted, context-appropriate supportive approaches.

## Figures and Tables

**Table 1:** Demographic characteristics of the included patients (*n* = 173), using frequency (*n*) and percent (%).

Parameter	Value	Frequency (*n*)	Percent (%)
Gender	Female	74	42.8%
	Male	99	57.2%
Environment	Urban	72	41.6%
	Rural	101	58.4%
Studies	Primary school/Middle school	54	31.2%
	High school/Post-secondary/Vocational school	89	51.4%
	University/Postgraduate studies	30	17.3%
Income	Medium threshold (50–100% of the national median line)	52	30.1%
	High threshold (>national median line or over 2× minimum wage in the economy)	121	69.9%

**Table 2:** Socio-economic status (SES-3 composite) by sex (*n* = 173), using frequency (*n*) and percent (%).

SES-3 Category	Female (*n*/%)	Male (*n*/%)	Total (*n*/%)
Low	33 (44.6%)	22 (22.2%)	55 (31.8%)
Medium	31 (41.9%)	55 (55.6%)	86 (49.7%)
High	10 (13.5%)	22 (22.2%)	32 (18.5%)

**Table 3:** Spearman correlations with 95% confidence intervals for depression, anxiety, stress severity symptoms, and dialysis adequacy.

Spearman r	Depression vs. Anxiety	Depression vs. Stress	Depression vs. spKt/V	Anxiety vs. Stress	Anxiety vs. spKt/V	Stress vs. spKt/V
r	0.3226	0.1789	0.1157	0.2862	−0.08782	−0.09958
95% confidence interval	0.1779 to 0.4537	0.02604 to 0.3235	−0.03848 to 0.2646	0.1388 to 0.4212	−0.2381 to 0.06663	−0.2493 to 0.05480
*p* value (two-tailed)	<0.0001 ****	0.0185 *	0.1294 ^ns^	0.0001 ***	0.2506 ^ns^	0.1924 ^ns^

Significance levels are marked as follows: * *p* < 0.05, *** *p* < 0.001, **** *p* < 0.0001, ns—not statistically significant.

**Table 4:** Spearman correlations with 95% confidence intervals for psychological measures, comorbidity burden, and dialysis duration.

Spearman r	Depression vs. CCI	Anxiety vs. CCI	Stress vs. CCI	spKt/V vs. CCI	Depression vs. Years of Dialysis	Anxiety vs. Years of Dialysis	Stress vs. Years of Dialysis
r	0.05526	0.05052	−0.01321	−0.1780	−0.06729	−0.01855	−0.1495
95% confidence interval	−0.09913 to 0.2070	−0.1038 to 0.2025	−0.1664 to 0.1406	−0.3227 to −0.02515	−0.2186 to 0.08715	−0.1716 to 0.1354	−0.2963 to 0.004099
*p* value (two-tailed)	0.4703 ^ns^	0.5092 ^ns^	0.8631 ^ns^	0.0191 *	0.3791 ^ns^	0.8086 ^ns^	0.0496 *

Significance levels are marked as follows: * *p* < 0.05, ns—not statistically significant.

**Table 5:** Multivariate logistic regression for depression, anxiety and stress.

Outcome	Area Under the ROC Curve		Variable	Odds Ratio	95% CI
Depression	AUC	0.6890	Age (in years)	1.003	0.9733 to 1.035
	Std. Error	0.04258	Sex	0.3010	0.1479 to 0.5974
	95% CI	0.6055 to 0.7724	CCI	1.111	0.9370 to 1.316
	*p* value	<0.0001	Years of dialysis	1.041	0.9498 to 1.141
Anxiety	AUC	0.5882	Age (in years)	0.9872	0.9555 to 1.020
	Std. Error	0.05013	Sex	0.7142	0.3334 to 1.530
	95% CI	0.4899 to 0.6865	CCI	1.132	0.9450 to 1.351
	*p* value	0.1038	Years of dialysis	1.057	0.9566 to 1.165
Stress	AUC	0.5624	Age (in years)	0.9929	0.9645 to 1.023
	Std. Error	0.04797	Sex	0.8289	0.4223 to 1.631
	95% CI	0.4684 to 0.6565	CCI	0.9559	0.8063 to 1.123
	*p* value	0.1959	Years of dialysis	0.9598	0.8713 to 1.051

## Data Availability

The data presented in this study are available from the corresponding author upon request. The data are not publicly available due to privacy restrictions.

## Abbreviations

The following abbreviations are used in this manuscript:

APA	American Psychological Association
BDI	Beck Depression Inventory
CCI	Charlson Comorbidity Index
CKD	Chronic Kidney Disease
DASS-21R	Depression, Anxiety and Stress Scale 21R
HADS	Hospital Anxiety and Depression Scale
HD	Hemodialysis
MMSE	Mini Mental State Examination
NICE	National Institute for Health and Care Excellence
NIH	National Institute of Health
PD	Peritoneal dialysis
SES-3	Socio-economic Scale
spKt/V	Single Pool Kt/V (urea clearance index)
STAI	State Trait Anxiety Inventory
